# Vulnerability Factors for the Psychiatric and Behavioral Effects of Cannabis

**DOI:** 10.3390/ph3092799

**Published:** 2010-08-26

**Authors:** Marco Bortolato, Valentina Bini, Simone Tambaro

**Affiliations:** 1 Department of Pharmacology and Pharmaceutical Sciences, University of Southern California, Los Angeles, CA 90033, USA; 2 Tourette Syndrome Center, University of Cagliari, Italy; E-Mail: valentina_bini@libero.it (V.B.); 3 Department of Neuroscience “Bernard B. Brodie”, University of Cagliari, Italy; 4 Neuroscience PharmaNess, S.C.A.R.L., Edificio 5, Parco Scientifico e Tecnologico della Sardegna, Località Piscinamanna, Pula, Cagliari, Italy; E-Mail: st_007@usc.edu (S.T.)

**Keywords:** cannabinoids, vulnerability, CB receptors

## Abstract

Cogent evidence shows that cannabis plays a variable role on behavioral regulation and the pathophysiology of most psychiatric conditions. Accordingly, cannabis has been alternatively shown to exacerbate or ameliorate mental symptoms, depending on its composition and route of consumption, as well as specific individual and contextual characteristics. The vulnerability to the psychological effects of cannabis is influenced by a complex constellation of genetic and environmental factors. In the present article, we will review the current evidence on the pharmacological, individual and situational factors that have been documented to affect the behavioral and psychiatric effects of cannabinoids.

## 1. Introduction

Recent epidemiological surveys have ascertained that marijuana and other hemp plant (*Cannabis sativa)* products are the most widely abused illicit substances in Western countries [1], and their consumption among minors and young adults is steadily rising [2,3]. One of the most worrisome implications of this scenario lies in the role of cannabis as a risk factor for the development of several staggering psychiatric conditions, including schizophrenia and bipolar disorder [4,5,6,7,8,9]. In striking contrast with this notion, however, several psychiatric patients are reported to use cannabis for its therapeutic properties [10,11,12,13]. Indeed, the effects of cannabis are extremely variable across different individuals, in relation to a broad array of vulnerability factors. The identification of these genetic and environmental elements is poised to become a critical goal for the enactment of preventative and therapeutic strategies for cannabis-related mental disorders; nevertheless, research on the biological underpinnings of susceptibility to cannabis is still in its preliminary stages and limited by the inadequacy of animal models to reproduce this inherently human aspect.

A promising approach to grapple with this nodal issue may be afforded by the analysis of the variations in emotional and cognitive effects of cannabinoids and their biological underpinnings. The literature on the behavioral outcomes of cannabis and related agents is fraught with apparent inconsistencies among different authors and laboratories. These discrepancies, often regarded as an important hindrance to our understanding of the psychotropic properties of cannabinoids, may prove a rich source of data to establish a theoretical framework to study the vulnerability for cannabis-induced outcomes.

## 2. The Endocannabinoid System

The hemp plant features about 80 terpenophenolic alkaloids, collectively named *phytocannabinoids*, [14] which are known to exert a number of medicinal properties (for a comprehensive review on the topic, see [15]). In spite of such a rich variety of ingredients, most psychotropic effects of cannabis are produced by one phytocannabinoid, Δ^9^-tetrahydrocannabinol (THC) [16]. Recent findings have revealed that other components, such as cannabidiol (CBD) and cannabichromene (CBC) may play a key role in the behavioral outcomes of cannabis and in the modulation of the psychological effects of THC [17]. The potential involvement of CBD in the psychological and behavioral effects of cannabis, however, remains elusive and is beyond the scope of this manuscript; for a thorough analysis of the issue, the interested reader is referred to the recent reviews by Zuardi *et al*. [18], and Scuderi *et al*. [19]. 

The isolation of THC and the development of its synthetic analogs led to the identification of two G_i/o_ protein-coupled receptors, respectively termed CB_1_ and CB_2_ [20,21]. Anatomical studies have revealed that these receptors display a highly divergent pattern of distribution throughout the organism: CB_1_ are highly abundant in the brain [22], and densely expressed in all the main regions that govern emotional and cognitive behavior, including prefrontal and cingulate cortex, amygdaloid complex, septo-hippocampal system, striatum, midbrain nuclei *etc*. [22,23,24,25,26]. Conversely, CB_2_ are prevalent in the periphery, and particularly in immune cells [27]. This topographical dichotomy has been recently tempered by a number of studies documenting the presence of CB_2_ receptors in several brain regions [28,29]. The ultrastructural localization of CB receptors in neurons is still partially unknown, but the bulk of evidence supports that CB_1_ are mainly located in presynaptic boutons [30,31], while CB_2_ have been generally detected in postsynaptic terminals [32,33]. This segregation seemingly suggests that CB_1_ and CB_2 _receptors may exert distinct actions in the regulation of brain functions, as well as the psychotropic effects of cannabinoids. Notably, the activation of presynaptic CB_1_ receptors has been shown to inhibit the opening of voltage-operated calcium channels and reduce the release of key neurotransmitters, including γ-aminobutyric acid (GABA) and glutamate [34,35,36,37,38]. The characterization of CB_1_ receptors in homo- and heterodimeric organizations, however, suggests that variable arrangements of this molecule may correspond to different functional roles [25,39,40,41,42].

Both CB_1_ and CB_2_ receptors are endogenously activated by a number of arachidonic acid derivatives (*endocannabinoids*), such as anandamide (*N*-arachidonoylethanolamine) [43] and 2-arachidonoylglycerol (2-AG) [44,45]. These two endocannabinoids are synthesized upon demand through enzymatic degradation of membrane phospholipids, and released into the synaptic space [46,47]. While 2-AG has been shown to be the main retrograde mediator in glutamatergic [48,49,50,51] and GABAergic synapses [52,53] via CB_1_ activation, anandamide may act as an activity-dependent modulator of monoamine neurotransmission in several brain regions [54,55,56]. Several findings appear also to indicate that endocannabinoids modulate the signaling of several neuropeptides and hormones [57,58,59,60,61,62,63]. This highly complex network of interactions (reviewed by López-Moreno *et al*. [64]) is reflected in the multifaceted modulatory effects of cannabis on the regulation of brain and behavioral functions. 

## 3. The Role of Cannabis in Behavioral Regulation and Psychiatric Disorders

### 3.1. Emotional and Cognitive Effects of Cannabis

In most users, inhalation or ingestion of modest amounts of cannabis increase sociability, relaxation, and creativity [65,66]; these anxiolytic and mood-enhancing properties are the main incentive to the recreational use of marijuana and other hemp products [67,68]. In contrast, a smaller contingent of individuals - particularly first-time users - experience a number of undesirable emotional effects after cannabis consumption, such as panic, phobic manifestations, dysphoria [69,70,71,72,73,74,75,76,77].

Notably, cannabis has been documented to induce psychotic symptoms, such as derealization, depersonalization, paranoia and auditory hallucinations [70]. In particular, these alterations are commonly manifested in two distinct nosographic entities [78]: (a) toxic psychosis, featuring euphoria, perceptual distortions, clouding of consciousness and cognitive deficits [79]; (b) functional psychosis, characterized by positive symptoms similar to those featured in schizophrenia (including bizarre and paranoid ideation), but no cognitive impairment. This disturbance is highly sensitive to antipsychotic agents and posited to reflect an underlying disorder in predisposed individuals. 

Acute cannabis consumption also triggers variable cognitive dysfunctions, encompassing short-term perceptual, psychomotor, attentional and mnemonic deficits [66,80,81,82], (for a thorough review on the topic, see Solowij and Pesa [83]). 

One of the key factors that have been shown to influence the differential responsiveness to cannabinoids is the dose of exposure. Particularly in first-time or non-habitual users, low dosages of THC or marijuana are generally conducive to euphoria, hilarity, relaxation and subtle perceptual changes; conversely, higher concentrations are known to elicit fear, agitation and psychotic manifestations, as well as attentional and mnemonic impairments [71,74,76,77,78,79,84,85,86].

The most stringent evidence concerning dose-dependent, bidirectional effects of cannabinoids on emotional responses comes from research in experimental rodents. Several studies have revealed that, in rats and mice, low doses of CB_1_ receptor agonists attenuate anxiety-like and depression-like behaviors, and enhance locomotion and exploratory activity; in contrast, elevated concentrations of cannabinoids are typically anxiogenic and aversive [87,88,89,90,91,92,93,94,95,96] and alter attentional processing, executive functions and working and recognition memory [97,98,99,100,101]. 

Interestingly, the behavioral impact of low doses of cannabinoids is posited to mimic the physiological effects of endocannabinoids. Accordingly, the pharmacological inhibition of reuptake and enzymatic degradation of anandamide has been shown to induce anxiolysis and antidepressant-like effects in animals [55,102,103,104]. Of note, endocannabinoids and low doses of cannabinoids have been found to support the encoding and retrieval of select types of memory, and to ameliorate cognitive functions in rodents [105,106]. While these findings point to the possibility that cannabinoids may exert biphasic effects also on select cognitive properties, evidence on this issue is still lacking. 

Prolonged consumption of cannabis, particularly in adolescence, is generally conducive to persistent affective and cognitive sequelae in adulthood [107]. These alterations include avolition and alexithymia [71,108,109,110], as well as impairments in informational processing, sustained and distributed attention, spatial working memory, verbal fluency, decision making and executive functions [76,81,111,112,113,114]. Interestingly, recent studies have correlated some of these deficits to abnormalities in cortical and hippocampal metabolism [115]. 

In addition to dose-related factors, other determinants play a key role in the behavioral effects of cannabis. For example, the relative composition in different ingredients (and particularly CBD and CBC) is likely to exert a profound influence on the emotional outcomes of this drug, in view of the anxiolytic properties of CBD [17,18,19]. Furthermore, the impact of cannabis is certainly modulated by a number of vulnerability factors, such as genetic background, age, gender *etc*. The available evidence on the role of these critical elements will be reviewed in the following chapter.

### 3.2. Anxiety- Spectrum and Mood Disorders

Although cannabis abuse and dependence are frequently comorbid with affective and anxiety disorders [9,86,116,117,118,119,120,121], very few studies have been focused on the pathophysiological link between these phenomena. While cannabis does not appear to play a conclusive role in affective and anxiety disorders [72,122,123,124], convergent lines of evidence show that its consumption can profoundly affect the severity and the clinical presentation of their symptoms. In conformity with the high variability in emotional outcomes observed in healthy individuals, however, cannabis has been shown to exert opposite effects in different clusters of patients. A number of studies have reported that this substance can precipitate the symptoms of anxiety-spectrum, bipolar and depressive disorders, and reduce the therapeutic efficacy of benchmark agents [7,125,126,127,128]. Conversely, several patients suffering from anxiety and mood disorders use cannabis as a relaxant and for self-therapeutic purposes [7,13,129,130,131,132,133]. In support of this last notion, anxiety disorders have been shown to predict later cannabis use disorder [74,134,135]. Furthermore, few clinical trials in the 1980’s have shown that nabilone - a cannabinoid analog approved in United Kingdom as an antiemetic treatment – has anxiolytic effects in psychiatric patients [136,137,138].

However, it is important to note that, while the initial consumption of cannabis can have relaxant effects in patients suffering from anxiety and mood disorders, its chronic use could actually exacerbate the symptoms of these illnesses by dampening the functions of the endocannabinoid system. 

The implication of endocannabinoid system in anxiety and depression has been also extensively shown by preclinical studies. While CB receptor ligands have been shown to induce variable effects on both anxiety- and depression-like behaviors in rodents (for reviews on this topic, see Vinod and Hungund [139], Bortolato and Piomelli [140], and Parolaro *et al.* [141]), blockade of anandamide reuptake and degradation elicits anxiolytic and antidepressant-like effects [55,102,104,105,142], suggesting a key role of this endocannabinoid in emotional regulation.

### 3.3. Psychotic Disorders

The relation between cannabis consumption and schizophrenia has been observed in many epidemiological surveys [4,143,144,145]. Several lines of evidence have convincingly shown that cannabis use is a risk factor for psychotic disorders [5,6,8]; in particular, longitudinal studies have documented that habitual cannabis consumption in adolescence leads to an increase in incidence of schizophrenia [122,146,147]. Indeed, cannabis consumption is frequently comorbid with first-episode schizophrenia [148]. 

This evidence is also supported by experimental studies, which found that cannabis products and THC can exacerbate positive and negative symptoms in schizophrenia patients [149,150]. Interestingly, administration of THC at high doses has been shown to cause endophenotypical alterations similar to those observed in schizophrenia patients, such as decrease in the P300 component of event-related potentials [151,152] and reduction of prepulse inhibition of the startle reflex [153,154]. 

Nevertheless, several schizophrenia patients have reported beneficial effects from cannabis consumption, such as the reduction of anxiety [10,11] and negative symptoms, such as affective flattening and anergia [155,156]. Furthermore, a small subset of psychotic patients motivate cannabis use as a self-therapeutic strategy to attenuate positive symptoms, such as auditory hallucinations and paranoia, or to countenance the side effects induced by antipsychotic medications [156,157].

Some lines of research suggest that the endocannabinoid system may exert an important modulatory role in the pathophysiology of psychosis [158]. For example, expression of CB_1 _receptors has been shown to be increased in prefrontal cortex, anterior and posterior cingulate cortex of schizophrenia patients [159,160]. However, this evidence has not been replicated by subsequent studies [161,162]. Anandamide levels are elevated in the CSF of first-episode schizophrenia patients [163], they have been found to be *inversely* correlated with psychotic symptoms [164]. Moreover, the excess of anandamide is reversed by typical antipsychotics [164], suggesting that this endocannabinoid may be instrumental for the homeostasis of dopamine neurotransmission. Interestingly, high cannabis use in first-episode schizophrenia patients has been shown to reduce anandamide levels; this down-regulation in anandamide signaling may in fact increase the risk for psychosis or precipitate its manifestations [163].

The neurobiological underpinnings of the role of endocannabinoids in schizophrenia are still largely elusive and may concern different systems, such as dopamine, glutamate and GABA, particularly in relation to their maturation during adolescence [165].

Taken together, this background supports the view that cannabinoids may play different roles in schizophrenia, probably in relation to the highly heterogenous nosological entities described by this category and potentially distinct functions of the endocannabinoid system in the pathophysiology of this disorder. 

The high variability of the impact of cannabis in schizophrenia-related alterations is also reflected in the numerous discrepancies on the effects of cannabinoids in rodents tested on the prepulse inhibition of the startle, a well-validated index of sensorimotor gating [166,167]. 

## 4. Vulnerability Factors

The seemingly contradictory scenario outlined in the previous section underscores the relevance of interindividual differences in the psychological and behavioral effects of cannabis in healthy individuals, psychiatric patients and animal models. The variations in the emotional and cognitive sequelae of cannabis are likely influenced by the interaction of drug-related characteristics (frequency and duration of use, dose consumed and relative concentrations of ingredients) and vulnerability factors, both intrinsic (including genetic vulnerability, age, gender, premorbid personality traits, *etc*.) and extrinsic (exposure to stress, concomitant use of other drugs *etc*.). 

### 4.1. Genetic Background

Although genetic components have been largely advocated as a factor of vulnerability for the emotional and cognitive effects of cannabis, research in this respect is still preliminary. McGuire and colleagues [168] found that healthy subjects with a positive family history of schizophrenia had a higher likelihood to develop hallucinations and other psychotic symptoms following cannabis consumption. 

The quest for genetic determinants has indicated a potential role of some of the polymorphic variants of the CNR1 gene, encoding for CB_1_ receptor protein [169]. Indeed, allelic variations of this gene have been associated with alcoholism and drug use [169,170,171,172], impulsivity [173], neuroticism [174] as well as different striatal responses to social stimuli [175,176]. A particular polymorphic variant of the CNR1 gene, featuring multiple Adenine-Adenine-Thymine (AAT) repeats and deemed to regulate the transcription of CB_1_ receptor, has been associated with different risks for depression in Parkinson disease patients [177] and a diagnostic cluster of schizophrenia [178,179], (for contrasting results, see [180]). Additionally, other alterations of CNR1 gene may act as a protective factor for schizophrenia, or induce a better pharmacological response to atypical antipsychotics [181]. 

In addition to allelic variations in CNR1 gene, other polymorphic variants and genetic factors have been linked to a different vulnerability for the role of cannabis in schizophrenia [182]. In particular, several lines of evidence have indicated that the Val^108^Met allelic variant Catechol-*O*-methyltransferase (COMT), which codifies for a high-activity variant of this enzyme, is associated with a higher risk of psychosis [183], particularly in conjunction with other predisposing environmental factors, such as psychosocial stress [184]. Recent evidence has also shown that different haplotypes of neuregulin 1 gene (*Nrg1*) have also been implicated in the sensitivity to the effects of cannabinoids in mice [185]. Finally, cannabis dependence has been associated with the C3435T single nucleotide polymorphism (SNP) of the gene ABCB1, encoding for the membrane transporter P-glycoprotein [186]. Specifically, the 3435 CC genotype (resulting in increased P-glycoprotein expression) was more common among cannabis-dependent patients than controls. Interestingly, the same polymorphism has been shown to influence the response to antipsychotic and antidepressant treatment [187,188], potentially suggesting a role of its variants in the psychiatric outcomes of cannabis [163].

### 4.2. Age and Gender

Adolescence has been shown to be an important vulnerability factor for cannabis-induced sequelae. For instance, juvenile age plays a key role in the development of attentional alterations following acute cannabis use, which are not commonly observed in adult users [189,190]. In line with this concept, the impairments in sustained attention in chronic adult users have been shown to increase in severity for users that initiated cannabis consumption before 15 years of age [191]. Early onset of cannabis consumption has been associated with a higher susceptibility to psychosis [4,122]. In view of the critical role of endocannabinoids in neurodevelopmental processes [158,192], it is likely that early consumption of cannabis may interfere with critical neurodevelopmental processes occurring throughout adolescence, including synaptic sprouting and pruning [193,194], myelination, changes in neurotransmitter concentrations *etc*. The consequences of exposure to non-endogenous cannabinoids in such a critical period may be particularly severe if associated with other factors, such as genetic or environmental determinants, and lead to profound alterations of the circuitry underpinning specific mental disorders. The role of age would then be particularly important for schizophrenia, in consideration of its neurodevelopmental nature [195].

Interestingly, some lines of clinical investigation have documented the existence of gender differences in the vulnerability to cannabis-mediated changes, with a higher severity of anxiety-like and depression-like manifestations in females [195,196], (for a general presentation of gender-related issues in cannabis-mediated effects, see Fattore and Fratta [197]).

The contribution of age and sex characteristics to the emotional effects of cannabis has been largely bolstered by animal experimentation. As reviewed by Rubino and Parolaro [107], the available preclinical evidence supports the possibility that adolescent exposure to cannabinoids and gender differences may affect emotional and cognitive behaviors in adulthood. For example, treatment of rats with cannabinoids during juvenile stages induce reductions in social interaction, novel object recognition, sensorimotor gating, and enhance depression-like behaviors in adulthood [98,198,199,200]. 

### 4.3. Environmental Contingencies

The anxiogenic and hallucinogenic effects of cannabis are more frequently observed in novel and stressful settings [201,202,203,204]. Early stress (particularly from child abuse or neglect) has been shown to interact with cannabis use in determining the risk of development of schizophrenia in adolescence [123,205,206,207]. In particular, cogent evidence points to the critical role of gene-environment interactions in the association between cannabis and psychosis [182].

Stressful environmental situations are also known to exacerbate the anxiety and aversive reactions associated with acute administration of cannabinoids in both humans and animals [140]. Conversely, environmental enrichment has been shown to enhance the rewarding properties of CB_1_ agonists in rodents [103]. It is important to note that the role of environmental factors on the outcomes of cannabis may also be mediated by its influence on the motivation to consume the drug, particularly in early adolescence [208]. 

### 4.4. Other Factors

In addition to the aforementioned determinants, the vulnerability for the psychiatric sequelae of cannabis is likely to include a number of other factors, such as the premorbid personality and concurrent use of other substances. Polydrug use is a common characteristic in cannabis users [209] and may certainly influence the pathophysiological trajectory of most behavioral and psychiatric effects of this substance.

Furthermore, the impact of cannabis is certainly dependent on the specific variety of substance consumed, with respect to the concentrations and the relative composition of psychoactive ingredients; indeed, the anxiolytic and antipsychotic effects of CBD have been postulated to largely contribute to the behavioral outcomes of cannabis [86,210,211]. 

## 5. Conclusions

While epidemiological and clinical evidence suggests that early and heavy cannabis abuse may enhance the risk of developing psychotic and possibly affective disorders, most users do not develop psychiatric conditions, and some may even use cannabis for self-medication purposes. Indeed, the relationship between cannabis and mental disorders is influenced by a wide set of determinants, related to individual and environmental characteristics, as well as the level, modality and regimen of drug exposure. The convergence of these factors may induce distinct alterations of the expression of CB receptors, as well as their functional link to downstream effectors (such as other neurotransmitter systems), thereby shaping the subjective effects of cannabis, as well as its role in the pathophysiology of mental disorders. Accordingly, chronic environmental stress has been shown to predispose to some untoward effects of cannabis and to induce changes in CB_1_ receptor expression in experimental animals [104,142,212,213]. 

On the other hand, chronic consumption of cannabis may facilitate the onset of psychiatric disorders by inducing a down-regulation of the endocannabinoid system. Most long-term sequelae of cannabis chronic consumption may not strictly result from the intrinsic behavioral effects of the drug, but mainly from the progressive neuroplastic processes enacted as a homeostatic response to the prolonged exposure to exogenous cannabinoids. Such mechanisms may lead to a progressive reduction of the synthesis and signaling of anandamide and 2-AG, as well as to a desensitization of CB receptors, possibly promoting the progression of certain psychiatric disorders. 

The recent availability of a positron emission tomography (PET) tracer for *in vivo* brain imaging of the CB_1_ receptor in humans and monkeys [214,215] affords a unique opportunity to test the specific role of this receptor in the vulnerability to the subjective responses to cannabis. Indeed, these ligands have already been employed to show gender differences in CB_1_ expression of healthy probands [216], and document an association between low CB_1_ receptor density and novelty-seeking personality trait [217]. These studies are paving the way for further work on a more detailed analysis of the neurobiological mechanisms by which several vulnerability factors may affect CB receptor responsiveness and influence the pathophysiology of cannabinoid-related mental disorders.
